# Performance Analysis of Clinical Chemistry Laboratory Using Sigma Metrics in the Total Testing Process at Dessie Comprehensive Specialized Hospital, Ethiopia

**DOI:** 10.1002/jcla.70134

**Published:** 2025-11-26

**Authors:** Zewudu Mulatie, Endris Ebrahim, Mihreteab Alebachew, Alemu Gedefie, Bruktawit Eshetu, Mihret Tilahun, Habtu Debash, Yeshimebet Kassa, Ermiyas Alemayehu, Tesfaye Gessese

**Affiliations:** ^1^ Department of Medical Laboratory Sciences, College of Medicine and Health Sciences Wollo University Dessie Ethiopia

**Keywords:** clinical chemistry laboratory performance, Ethiopia, sigma metrics

## Abstract

**Background:**

Clinical laboratory test results in clinical decisions have become a fundamental element of clinical practice, with laboratory results influencing approximately 70% of decisions. The sigma metrics method is used to evaluate all stages of the testing process in clinical chemistry laboratories.

**Objective:**

This study aims to assess the performance of the clinical chemistry laboratory using sigma metrics across the total test process at Dessie Comprehensive Specialized Hospital.

**Methods:**

A cross‐sectional study was conducted from July 1, 2024 to September 30, 2024. The study included all the eligible laboratory samples and corresponding test requests during the study period, selected using consecutive sampling techniques. Data for each variable were collected using a prepared checklist and record format by trained laboratory professionals. The data were entered into EPI Data version 3.1 and analyzed using Stata version 17. Laboratory performance was evaluated using the sigma metrics.

**Result:**

The overall performance of the clinical chemistry laboratory was inadequate, with an overall sigma value of 2.9. The laboratory showed marginal performance in the analytical phase (3.5 sigma value) and low performance in the pre‐analytical and post‐analytical phases (2.8 and 2.9 sigma, respectively). However, most clinical chemistry parameters demonstrated satisfactory sigma metric values (≥ 3).

**Conclusion:**

The hospital administration should provide training for all staff involved in laboratory sample collection and processing, focusing on quality control, reagent handling, and adherence to SOPs, along with continuous performance monitoring.

## Graphical abstract

Clinical laboratory test results guide ~70% of clinical decisions. Using sigma metrics, the clinical chemistry laboratory at Dessie Comprehensive Specialized Hospital showed overall inadequate performance (2.9 sigma), with marginal performance in the analytical phase (3.5) and low performance in the pre‐ and post‐analytical phases (2.8 and 2.9). Strengthening staff training in sample collection and processing is essential to improve laboratory quality.

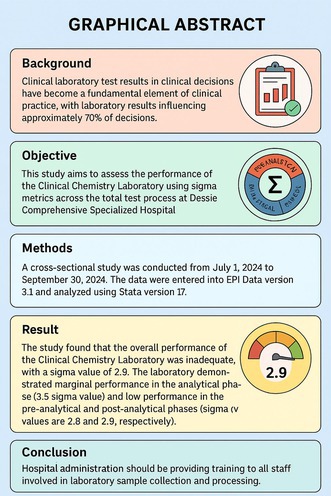

**Abbreviations**
CLIA: clinical laboratory improvement amendment
IPD: inpatient department
ISO: international organization for standardization
MRN: medical record number
OPD: outpatient department
QIs: quality indicators
TAT: turnaround times
TTP: total testing process
WHO: World Health Organization

## 1 Introduction

### 1.1 Background

According to research, the use of clinical laboratory test results in clinical decision making has become an essential component of modern medicine [1, 2]. Approximately 70% of clinical decisions involving hospitalization, admission, prescription, and discharge are dependent on laboratory results [1, 3, 4]. In clinical medicine, screening, diagnosis, and therapy monitoring for many diseases and disorders are based on clinical chemistry laboratory results. Therefore, the quality of laboratory services plays a significant role in the total testing process (TTP) to ensure the release of accurate, precise, and timely results for patient care [5, 6, 7]. The total laboratory testing process is a complex system that begins and ends with the patient, starting from the medical decision to order a test and concluding with the communication of the results. It comprises three main phases: the pre‐analytical, analytical, and post‐analytical [8, 9].

Patient safety is a major global public health concern. According to a World Health Organization (WHO) report, the global burden of unsafe care is estimated at 421 million cases annually, making it one of the ten leading causes of death and disability worldwide [10]. Diagnostic errors remain a significant issue, with studies reporting a prevalence of around 3.6% in certain contexts [11]. In the United States, this figure rises to approximately 5% among outpatient cases [12, 13]. Moreover, research highlights serious challenges in low and middle‐income countries, where an estimated 134 million adverse events and about 2.6 million deaths occur each year due to medical errors. Notably, South Asia and Western Sub‐Saharan Africa experience the highest mortality rates, with approximately 1.9 million and 1.2 million deaths, respectively [14, 15].

The impact of laboratory errors can be substantial but can be reduced by up to 15% when managed effectively worldwide [13, 14]. Such errors contribute to various clinical challenges, including delayed diagnoses, unnecessary repeat tests, and misdiagnoses, leading to issues such as over‐medication, under‐medication, inappropriate treatments, increased healthcare costs, reduced patient satisfaction, and even serious consequences like injury or death [16, 17]. As a result, ensuring high‐quality laboratory services has become a major priority for governments worldwide [18, 19]. In Africa, the burden of laboratory errors is higher than in other continents due to inadequate healthcare infrastructure, insufficiently trained employees, poor management systems, ineffective control measures, equipment shortages, weak government policies, low worker motivation, and widespread poverty [20, 21]. Consequently, most clinical laboratory services in Africa remain below standard, and the participation of laboratories in accreditation processes is below the expected level [22]. According to the ISO 15189 report, the performance of African laboratories is poor. Similarly, most laboratories in Ethiopia are not accredited, and their quality falls below the standard. Despite this, laboratory errors have received little attention and remain poorly understood [22, 23, 24, 25].

Errors are part of human nature; however, the ability to create solutions and discover better options is also inherent. Despite these facts, recently several laboratories around the world have applied principles of sigma metrics in all phases of TTP to improve quality, decrease the number of defects, improve the registration process, reduce patient wait times, lower costs, and meet customer expectations [26]. In the pre‐analytical phases, sigma metrics can be used to improve the quality of information on requisitions, specimen collection, patient identification, and transportation. In the analytical phase, it can help reduce laboratory testing errors, misinterpretation, misreading, and misjudging of the results [27]. Although quality essentials and QC programs were established to ensure quality service by identifying defects, this approach alone is insufficient and does not provide a direct and integrated assessment of the testing process's performance in laboratories [3, 28]. Therefore, sigma metrics are important tools for assessing laboratory performance to achieve the intended goals [28].

Sigma metrics is one of the tools to quantify the performance of a laboratory process as a rate of defects‐per‐million opportunities [29]. It is more effective for evaluating laboratory errors than relying on the number of defects alone because sigma metrics analysis is used to assess the quality of laboratory TTP by providing error correction mechanisms and performance improvement methodologies such as the define, measure, analyze, improve, control model. Additionally, sigma metrics help determine the number of quality controls needed in a laboratory [30, 31]. Evidence indicates that achieving sustainable laboratory performance in African countries remains challenging, with substantial quality gaps. Most clinical laboratories continue to provide suboptimal services, and the quality of these services is poor [25, 32]. Despite this, few studies have assessed the overall distribution of clinical chemistry laboratory errors, and even fewer studies have evaluated process performance using the sigma metric in Ethiopia. Therefore, the present study aims to assess the performance of the clinical chemistry laboratory across the TTP using sigma metrics, and to evaluate the performance of the chemistry analyzer (Dimension EXL 200 Integrated Chemistry System analyzer) based on sigma metrics.

## 2 Materials and Methods

### 2.1 Study Area, Design, and Period

A cross‐sectional study was conducted at the Dessie Comprehensive Specialized Hospital clinical chemistry laboratory from July 1, 2024 to September 30, 2024. The Dessie Comprehensive Specialized Hospital laboratory comprises several sections, including phlebotomy room, hematology, parasitology, clinical chemistry, urinalysis, serology, emergency, and microbiology. Among these, the clinical chemistry laboratory is the principal section, performing routine clinical chemistry tests, hormone tests, and electrolyte analyses. The clinical chemistry laboratory processes approximately 50,000 samples annually.

### 2.2 Population

All phlebotomists, laboratory professionals and laboratory test requests with their respective blood samples for analysis were considered as source populations.

### 2.3 Inclusion and Exclusion Criteria

#### 2.3.1 Inclusion Criteria

Test requests with samples for clinical chemistry laboratory tests were included.

#### 2.3.2 Exclusion Criteria

Tests requested with samples for non‐routine clinical chemistry laboratory tests were excluded. Additionally, tests that experienced inconsistent reagent supplies were also excluded.

### 2.4 Variables

#### 2.4.1 Dependent Variables

Clinical chemistry laboratory performance.

#### 2.4.2 Independent Variables

Sample collection site, work shift and adherence to SOP.

### 2.5 Operational Definitions

**Clinical laboratory error**: Any mistake that occurs in any phase of the total testing process.

**Pre‐analytical errors**: Mistakes occurring before sample analysis.

**Analytical errors**: Mistakes that occur during the testing.

**Post‐analytical errors**: Mistakes occurring after analysis or testing [33].

**Total error**: The combined effect of all possible errors throughout the total testing process [34].

**Critical values**: Results that exceed or are below the reference range and require immediate medical attention.

**Hemolysis**: The destruction of red blood cells that causes visibly red plasma in a tube.

**Lipemic**: Plasma with a visible milky appearance due to excess lipids.

**Clotted sample**: A specimen containing solid fibrin clots that may block analyzer probes [35].

**Insufficient sample**: A specimen with a volume below the minimum required for analysis.

**Over filled sample**: A specimen with volume exceeding the acceptable limit for analysis.

**Sample delayed**: A specimen kept at room temperature for more than 2 h before analysis.

**Turnaround time**: The time interval between sample collection and results released to the physician.

**Phlebotomist**: A laboratory or other health professional who is responsible for collecting blood specimens.

**Work shift**: A scheduled working period in the laboratory; in this study, two shifts were used (8:00–12:30 AM and 1:00–5:00 PM).

**Total allowable error**: The maximum variation from the true value of an assay, which will be taken from Clinical Laboratories Improvement Amendments (CLIA) guidelines [36].

**Target or true value**: The best available estimate of the parameters analyzed, which is obtained from the manufacturer or reference method.

**Bias**: The systematic difference between the observed mean of test results and the accepted reference or peer mean.

**Tolerance limit**: An acceptable range using target value and total allowable error (TV + TEa).

**Total observed error**: The total variation of a test result from the true value, incorporating both random and systematic errors.

**Sigma metrics**: Is the maximum number of SD closest to the tolerance limit from the mean of the assay.

A test is said to be unacceptable if the total observed error is greater than the total allowable error. In this study performance is evaluated based on the sigma value listed in the table below [37, 38] (Table 1).

**TABLE 1:** Sigma value interpretations table.

Sigma value	Defect per million opportunities	Yield (%)	Level
1	690,000	30.9	Unacceptable
2	308,537	69.1	Poor
3	66,807	93.3	Marginal
4	6210	99.4	Good
5	233	99.98	Excellent
6	3.4	99.99966	World‐class

**Overall performance of the test is unacceptable**: The average sigma value will be less than or equal to three [38].

**Overall performance of the test is acceptable**: The average sigma value will be greater than three [38].

**Quality goal index:** The ratio of bias to CV used to identify the primary cause of a low sigma value. A QGI > 1.2 indicates inaccuracy, a QGI between 0.8 and 1.2 suggests both imprecision and inaccuracy, and a QGI < 0.8 indicates imprecision.

### 2.6 Sample Size Determination and Sampling Technique

The study included all the eligible clinical chemistry laboratory samples and the corresponding test requests, which were requested during the data collection period. All test requests ordered for clinical chemistry laboratory tests during the study were included by using consecutive sampling techniques.

### 2.7 Data Collection and Laboratory Methods

Data was collected using the standardized pre‐tested checklist to evaluate the TTP of errors for the clinical chemistry laboratory. The checklist was prepared using variables from different studies, the International Federation of Clinical Chemistry checklist for accreditation of general laboratories, and clinical chemistry laboratories [17, 39, 40]. After preparing the checklist, data collectors, and supervisors were selected.

All of the data collectors were laboratory professionals who took job training in laboratory quality management systems. They trained on how to collect all the necessary data in the assessment of the completeness of the standard laboratory request forms (such as patient age, gender, signature of physicians, location, date, and authorized request formats), in the assessment of specimen quality, analytical and post‐analytical QIs based on the checklist.

Data collectors were assigned to sample collection and clinical chemistry sections of the laboratory. Subsequently, they collected all the required data based on the checklist provided by observation in all phases of TTP. Additionally, face‐to‐face interviews were conducted to gather socio‐demographic information from phlebotomists and laboratory personnel. Data on pre‐analytical variables, specifically related to laboratory specimens, were collected through observation by assessing each laboratory request and patient sample as soon as they were transported to the clinical chemistry laboratory. The post‐analytical variables were collected after the end of analysis and every variable in the checklist was entered as a record.

### 2.8 Quality Control

The quality of the data was assured by using a standardized observational checklist. The pre‐test was conducted on 40 samples with their corresponding test requests for the clinical chemistry laboratory to assess the feasibility and validate the study tools at Borumeda General Hospital. Then after, the checklist was modified based on the pre‐test results. Moreover, the quality of the data was assured by checking the completeness of the checklist on the spot by the data collectors while collecting relevant data at each phase of the clinical chemistry laboratory testing process. A supervisor provided daily feedback and corrections to the investigators throughout the data collection process. In addition, the completeness, accuracy, and clarity of the collected data were checked carefully regularly.

### 2.9 Data Analysis and Interpretation

Data was coded and entered into EPI data version 3.1 to check for completeness and accuracy before being exported to Stata version 17 for analysis. Descriptive statistics were used to illustrate the overall distribution of errors in the clinical chemistry laboratory.

Each phase's output was monitored, with errors and defects counted to determine the rate per million. This data was then translated into sigma metrics using a standard conversion table. The second aspect involved assessing the analytical quality control performance of routine renal and liver function tests, as well as electrolyte parameters from the Dimension EXL 200 Integrated Chemistry System. This evaluation was conducted using online sigma calculators, specifically the Defects Per Million calculator (available at https://www.westgard.com/six‐sigma‐calculators.htm) and the process sigma calculator (available at https://www.isixsigma.com/process‐sigmacalculator). Furthermore, the Quality Goal Index (QGI) ratio was computed using the formula: Bias%/(1.5 × CV%) to identify the reasons behind low sigma values across various parameters. A summary of the sigma metrics for each laboratory parameter was presented in a table.

## 3 Result

### 3.1 General Information

During the study period, a total of 9826 tests were requested. Of these, more than half were routine clinical chemistry tests (54.80%), followed by thyroid hormonal tests (26.57%). Among the total tests requested, 5093 (51.83%) were from the outpatient department (OPD) and 1625 (16.54%) were from maternal and child health (MCH) services. Additionally, more than half of the samples, 5661 (57.61%), were requested during the morning work shift. Furthermore, 7074 (72.0%) of the total samples were collected by laboratory professionals. All samples were recorded manually (Table 2).

**TABLE 2:** General information for clinical chemistry laboratory at Dessie Comprehensive Specialized Hospital, 2024.

Variables	Frequency	Percentage
Total test request assessed	9826	100
Test request for routine clinical chemistry	5385	54.80
Test request for electrolyte	1834	18.67
Test request for thyroid	2607	26.53
Sample requested site		
OPD	5093	51.83
IPD	1013	10.31
Emergency	605	6.16
MCH	1625	16.54
Unknown addresses	1490	15.16
Sample collection shift		
First (morning)	5661	57.61
Second (afternoon)	4165	42.39
Type of sample collectors		
Laboratory professionals	7074	72.0
Non‐laboratory healthy professionals	2751	28.10
Recording system		
Manually	9826	100
Laboratory information system	0	0

### 3.2 The Overall Prevalence of Errors and Performance Levels by Sigma Metrics in Clinical Chemistry Laboratory

In this study, 390,836 quality indicators were evaluated, revealing that 36,246 clinical chemistry laboratory errors occurred, which corresponds to a percentage of 9.27%. The distribution of these errors was as follows: 27,508 (75.89%) errors were found in the pre‐analytical phase, 664 (1.83%) in the analytical phase, and 8074 (22.28%) in the post‐analytical phase. Furthermore, the overall sigma value for the chemistry laboratory was 2.9, with the mean sigma values of 2.8 for the pre‐analytical, 3.5 for the analytical, and 2.9 for the post‐analytical phase.

### 3.3 The Sigma Metrics Levels of Missed Information on Test Requests

The sigma values for inappropriate and unauthorized requests, missed MRN and test ordered and handwriting illegible were greater than three. However, the average sigma value of the request form completeness was less than three (Table 3).

**TABLE 3:** Sigma metrics performance levels of missed information and related to laboratory test requests at the Dessie Comprehensive Specialized Hospital, Northeast Ethiopia, 2024.

Missed information		% of error	DPMO	Sigma value
Variables	No. of error			
Appropriate and authorized requests	398	4.1	40,505	3.3
MRN	145	1.5	14,757	3.7
Patient age	1757	17.9	178,811	2.5
Sex of patient	1485	15.1	151,130	2.6
Signature of the physician	2868	29	291,879	2.1
Clinical history of the patient	9826	100	1,000,000	1.0
Patients address	6495	66.1	661,001	1.1
Name of sender address/ward	794	8.1	80,806	2.9
Date of ordered	1320	13.4	134,337	2.7
Test ordered	0	0	0	> 6.0
Time of sample collection	853	8.7	87,218	2.9
Handwriting legible	564	5.7	57,399	3.1
Total	26,505	22.5	224,786	2.3

Abbreviations: DPMO, defect per million opportunities; MRN, medical record number.

### 3.4 The Sigma Metrics Levels of Pre‐Analytical Phase Related With Specimen Quality, Collection, Preparation, Storage, and Transportation in the Clinical Chemistry Laboratory

In the assessment of the quality of specimens, 338 (3.4%) specimens were unsuitable for analysis. The sigma values for hemolyzed, clotted, icteric, lipemic, incorrectly labeled, and insufficient samples were greater than 3.7. However, the overall pre‐analytical performance level was less than three sigma values (Table 4).

**TABLE 4:** Sigma metrics levels in pre‐analytical phases related to specimen quality, collection, preparation, storage, and transportation, Dessie Comprehensive Specialized Hospital, Northeast, Ethiopia, 2024.

Variables	No. of error	% of error	DPMO	Sigma value
Hemolyzed samples	134	1.4	13,637	3.8
Clotted samples	56	0.6	5699	4.1
Icteric	12	0.1	1221	4.6
Insufficient volume	126	1.3	12,823	3.8
Lipemic	17	0.2	1730	4.5
Specimen collected at the wrong time	47	0.5	4783	4.1
Incorrectly labeled specimen	156	1.6	15,876	3.7
The test tube broke in the centrifuge	0	0	0	> 6
Incorrect containers	0	0	0	> 6
Delayed samples	116	1.2	11,805	3.8
Wrong sample transportation	21	0.2	2137	4.4
Sample lost	11	0.1	1119	4.6
Request lost	16	0.2	1628	4.5
Patient identified improperly	97	1.2	11,934	3.8
Incorrect tourniquet application time	141	1.7	17,347	3.7
Specimen collected for unavailable test	32	0.3	3257	4.3
Total	1003	0.7	6520	4.0
Sample unsuitable for analysis	338	3.4	34,399	3.4
Total pre‐analytical errors	27, 508	10.1	101,224	2.8

Abbreviations: DPMO, defect per million opportunities; *N*, total frequency.

### 3.5 Sigma Metrics Levels of Analytical Phase in Clinical Chemistry Laboratory

Of the estimated total 2508 daily IQC, 48 (1.91%) were not performed. The sigma value for preventive maintenance not performed and nonlinear results were released without retesting was less than three. In addition, the sigma values for IQC performed as expected and IQC passed were greater than three. The overall sigma value for the analytical phase was 3.5 (Table 5).

**TABLE 5:** The sigma metric levels of analytical phase at Dessie Comprehensive Specialized Hospital, Northeast Ethiopia, 2024.

Variables	No. of error	% of error	DPMO	Sigma value
Daily IQC not performed	48	1.9	19,139	3.6
IQC result failed	72	2.9	29,268	3.4
Preventive maintenance not performed	14	17.3	172,840	2.5
Equipment mal‐functionality observed	4	6.1	60,606	3.1
Reference range unavailable for parameters	0	0	0	> 6
Electric power inconsistence during analysis	246	2.6	26,290	3.5
Non‐linear results were released without retesting	9	25.7	257,143	2.2
Working reagents prepared incorrectly	3	3.3	32,609	3.4
Reagents used after their expiration date	0	0	0	> 6
Inappropriate reagent storage condition	0	0	0	> 6
Reagent stock out during analysis	263	2.8	28,107	3.5
Questionable results were not retested	5	8.1	80,645	2.9
Methods not updated upon new reagent	0	0	0	> 6
Calibration failed	0	0	0	> 6
Total	664	2.6	26,155	3.5

### 3.6 Sigma Metrics Level of Post‐Analytical Phase

Sigma values for lack of critical results communication with physicians (1.9) and result release without verification (0.7) were less than three. The mean sigma value for the post‐analytical phase was less than three (2.9) (Table 6).

**TABLE 6:** Sigma metrics performance level of post analytical phase at Dessie Comprehensive Specialized Hospital, Ethiopia, 2024.

Variables	No. of error	% of error	DPMO	Sigma value
Critical values were not communicated to the physician immediately	123	97.6	976,190	1.9
Results released without result verification	7465	79.8	7,977,798	0.7
Test results unrecorded	48	0.5	5130	4.1
Results released without TAT	256	2.7	27,359	3.0
Result printouts attached to the wrong request	14	0.1	1496	4.5
Result reported without standard unit	65	0.7	6947	4.0
Samples were not retained/stored as the policy	51	0.5	5450	4.1
Laboratory results lost	13	0.1	1389	4.5
Results reported with incorrect standard unit	0	0	0	> 6
Results reported without reference range	34	0.4	3634	4.2
The result reported by unauthorized personnel	5	0.1	534	4.8
Total	8074	8.6	86,172	2.8

Abbreviations: DPMO, defect per million opportunities; TAT, turnaround time.

### 3.7 Location of Sample Collection and Shift With Pre and Post‐Analytical Errors

From the total of 9826 clinical chemistry requests evaluated, there were 771 (7.84%) and 967 (9.84%) test requests at unknown address and morning shift that were not filled for patients' age, respectively. From 338 rejected specimens, the frequency of hemolyzed samples was 37 (0.38%) and 89 (0.91%) at emergency section and afternoon shift, respectively. Out of 256 results released out of the expected TAT, 95 (1.02%) results were reported to unknown address (Table 7).

**TABLE 7:** Association of patient location and shift with pre‐analytical and post‐analytical errors at Dessie Comprehensive Specialized Hospital, 2024.

Variables	Location of patients (Clinic/Ward)					Shift	
	OPD	IPD	Emergency	MCH	Unknown	Morning	Afternoon
Patient age missed	234 (2.38)	216 (2.20)	330 (3.35)	206 (2.09)	771 (7.84)	967 (9.84)	790 (8.04)
Gender of patient missed	329 (3.35)	205 (2.09)	412 (4.19)	46 (0.47)	493 (5.02)	804 (8.18)	681 (6.93)
Signature of the physicians missed	959 (9.76)	180 (1.83)	388 (3.95)	445 (4.53)	896 (9.12)	1570 (15.98)	1290 (13.21)
Date of ordered missed	223 (2.27)	75 (0.77)	427 (4.35)	326 (3.32)	269 (2.74)	848 (8.63)	472 (4.80)
Hemolyzed specimen	28 (0.28)	31 (0.32)	37 (0.38)	16 (0.16)	22 (0.22)	45 (0.46)	89 (0.91)
Mislabeled specimen	43 (0.44)	12 (0.12)	52 (0.53)	20 (0.20)	29 (0.29)	41 (0.42)	115 (1.17)
Specimen rejection	132 (1.34)	72 (0.73)	20 (0.20)	42 (0.43)	72 (0.73)	166 (1.69)	172 (1.75)
Unrecorded results	9 (0.09)	5 (0.05)	27 (0.29)	1 (0.01)	6 (0.06)	12 (0.13)	36 (0.38)
Results released out of TAT	70 (0.75)	34 (0.36)	12 (0.13)	45 (0.48)	95 (1.02)	169 (1.81)	87 (0.93)

### 3.8 Sigma Value and Quality Goal Index of Clinical Chemistry Tests in Dimension EXL 200 Integrated Chemistry System Analyzer

Using clinical laboratory improvement amendment guidelines as a source of TEa, the sigma metric values of 20 clinical chemistry parameters were calculated. Based on the measured values, 6 analytes (urea L1 and 2, glucose L2, TBIL L1, DBIL L1, sodium L1, and potassium L1) had less than 3 sigma values and the remaining analytes were found to be greater than 3 sigma values. The lowest sigma value (1.04) was observed in urea (level‐I). In addition, the calculated QGI showed the presence of imprecision, inaccuracy and both imprecision and inaccuracy. It was found that the majority of the poor performance of analytes in the sigma value was due to the imprecision of the QC (Table 8).

**TABLE 8:** Total allowable error (TEa), CV, bias, sigma value, and QGI of the clinical chemistry tests in Dimension EXL 200 Integrated Chemistry System machine.

Parameters	IQC level	TEa%	Our EQA mean	Peer group mean	CV%	Bias%	Sigma level	QGI	Problem
ALT	L1	20	160.2	162.4	4.45	−1.35468	4.8	0.20306	Imprecision
	L2	20	160.2	162.4	4.04	−1.35468	5.29	0.22333	Imprecision
AST	L1	20	220.8	207.24	3.46	6.543138	3.89	1.261444	Inaccuracy
	L2	20	220.8	207.24	3.25	6.543138	4.14	1.343953	Inaccuracy
ALP	L1	30	192.6	189.84	4.79	1.453856	5.96	0.2024	Imprecision
	L2	30	192.6	189.84	4.26	1.453856	> 6	—	—
UR	L1	9	51.79	51.38	7.86	0.794083	1.04	0.06732	Imprecision
	L2	9	51.79	51.38	6.73	0.794083	1.22	0.078715	Imprecision
UA	L1	17	5.98	5.844	2.06	2.327173	> 6	—	—
	L2	17	5.98	5.844	1.53	2.327173	> 6	—	—
CRE	L1	15	3.776	3.83	3.63	−1.40992	4.52	0.25901	Imprecision
	L2	15	3.78	3.83	2.08	−1.40992	> 6	—	
GLU	L1	10	100.2	100.82	3.19	−0.61496	3.33	0.1285	Imprecision
	L2	10	100.2	100.82	3.64	−0.61496	2.92	0.11258	Imprecision
TC	L1	10	136.2	145.86	3.40	−6.62279	4.89	1.29891	Inaccuracy
	L2	10	136.2	145.86	3.84	−6.62279	4.33	1.15073	Imprecision and inaccuracy
TG	L1	15	1.538	1.541	2.94	−0.19468	5.17	0.04414	Imprecision
	L2	15	1.538	1.541	3.45	−0.19468	4.40	0.03759	Imprecision
HDL	L1	20	41.4	49.86	6.40	−16.9675	5.78	1.76656	Inaccuracy
	L2	20	41.4	49.86	3.20	−16.9675	> 6	—	—
LDL	L1	20	68.2	71.94	5.85	−5.19878	4.31	0.59254	Imprecision
	L2	20	68.2	71.94	4.57	−5.19878	5.51	0.75889	Imprecision
TP	L1	10	5.74	5.58	1.42	−2.867384	> 6	—	—
	L2	10	5.74	5.58	2.43	−2.867384	5.3	0.78776	Imprecision
ALB	L1	10	29.2	32.58	3.59	−10.3745	5.68	1.92472	Inaccuracy
	L2	10	29.2	32.58	4.57	−10.3745	4.46	1.51453	Inaccuracy
TBIL	L1	20	3.88	3.66	6.73	6.010929	2.08	0.595273	Imprecision
	L2	20	3.88	3.66	2.14	6.010929	> 6	—	—
DBIL	L1	20	0.47	0.59	21.12	−20.339	1.91	0.64206	Imprecision
	L2	20	0.47	0.59	7.68	−20.339	5.25	1.76524	Inaccuracy
Sodium	L1	5	141.8	140.14	1.69	1.1845	2.26	0.4658	Imprecision
	L2	5	141.8	140.14	1.05	1.1845	3.63	0.7536	Imprecision
Potassium	L1	5	3.64	3.728	2.47	−2.3605	2.98	0.6365	Imprecision
	L2	5	3.64	3.728	1.66	−2.3605	4.43	0.9468	Imprecision and inaccuracy
Chloride	L1	5	94.6	99.4	2.59	−4.8290	3.79	1.2384	Inaccuracy
	L2	5	94.6	99.4	1.91	−4.8290	5.15	1.6851	Inaccuracy
LDH	L1	20	351.2	360.88	3.64	−2.68233	> 6	—	—
	L2	20	351.2	360.88	2.40	−2.68233	> 6	—	—
Total CK	L1	30	310.2	303.62	2.43	2.167183	> 6	—	—
	L2	30	310.2	303.62	1.46	2.167183	> 6	—	—

Abbreviations: ALB, albumin; ALP, alkaline phosphatase; ALT, alanine transaminase; AST, aspartate transaminase; CK, creatinine kinase; CRE, creatinine; CV, coefficient of variation; DBIL, direct bilirubin; EQC, external quality control; GLU, glucose; HDL, high density lipoprotein; IQC, internal quality control; LDH, lactate dehydrogenase; LDL, low density lipoprotein; QC, quality control; QGI, quality goal index ratio; TBIL, total bilirubin; TC, total cholesterol; TEa, total allowable error; TG, triglyceride; TP, total protein; UA, uric acid; UR, urea.

## 4 Discussion

The current study aimed to assess the performance of the clinical chemistry laboratory in managing errors in TTP. The findings revealed that the overall performance of the clinical chemistry laboratory was poor, with a sigma value of 2.9. This result was similar to a study conducted in Gondar, Ethiopia (2.2 sigma value) [41]. However, our laboratory's performance was worse than that of other laboratories reported in studies conducted in Tehran, Iran [1], Guangdong, China [42], India [43], Mysore, India [44] and Turkey [3], where performance levels exceeded three sigma values. This discordance may be attributed to variations in QIs, test ordering systems, laboratory facilities, and the skill and experience of health professionals. Notably, the laboratories in those studies included fewer comprehensive QIs and utilized an electronic system for ordering all tests, unlike the current study.

The poor performance of our laboratory could be due to a lack of adherence to standard operating procedures during pre‐examination, examination, and post‐examination phases, as well as sample collection by non‐laboratory health professionals, electrical fluctuations, and a weak result reporting system. Specifically, our study found that electrical fluctuations, sample collection by non‐laboratory professionals, and non‐compliance with standard operating procedures accounted for 2.63%, 28%, and 39% of errors, respectively. Additionally, factors such as inadequate infrastructure, limited management skills, low staff motivation, and insufficient training may have contributed to the poor performance. These challenges could hinder future laboratory accreditation by reducing its recognition. Therefore, hospital leadership must take proactive measures to address these issues and enhance all aspects of laboratory operations. Error rates can be minimized through simple yet effective strategies, such as implementing robust policies to enforce protocols, developing a Laboratory Information System, providing professional training, adopting suitable technologies, and regularly monitoring quality improvement processes.

In the current study, the pre‐analytical phase had the mean sigma value below the acceptable threshold of three sigma levels. This underperformance could be attributed to limited experience, negligence, and insufficient focus on this phase. In contrast, the laboratory's performance in the analytical and post‐analytical phases was marginal (3.5 sigma) and poor (2.9 sigma), respectively. The suboptimal performance in the analytical phase may be linked to factors such as a shortage of trained personnel, insufficient reagents, inadequate preventive maintenance, a small sample size, and a lack of verification processes and awareness regarding critical value checks. Similarly, poor performance in the post‐analytical phase may result from inadequate communication of critical test results to physicians, insufficient result verification, and failure to release results within the established turnaround time (TAT). Additional contributing factors include increased workload, weak implementation of laboratory policies, ineffective reporting systems, limited infrastructure, and power supply instability.

In this study, the sigma value for the incomplete filling of test request forms was below the acceptable limit (2.3). This finding was similar to a study conducted in Gondar, Ethiopia, which reported a sigma value of less than 3 [41]. In contrast, our performance was worse than the studies conducted in India [45] and Romania [42], which reported optimal performance (> 3). This variation could be attributed to a lack of experience and the variability of physicians attending the patients at a given site, as most physicians in the study settings were medical students.

According to the current study, the laboratory achieved marginal performance (3.4 sigma value) in specimen quality. This indicates that our laboratory's performance in sample quality is worse than that reported in a study conducted in eastern India (> 4 sigma value) [46]. The poor performance of our laboratory may be due to a lack of adherence to SOPs, reliance on manual recording systems, work overload, and sample collection by untrained professionals.

In the other scenario, the laboratory achieved marginal to good performance (3.7–4.1 sigma value) for hemolysis, incorrect labeling, clotting, and incorrect volume. However, while this performance is within the acceptable limit, it is not considered world‐class. Studies conducted in India [45], China [42], Romania [42], and the United States [47] reported better performance (ranging from very good to world‐class) for these variables. A possible explanation for this lower performance may be the improper application of a tourniquet during sample collection, inadequate mixing of blood with anticoagulant, negligence, and improper sample transportation conditions.

In this study, the performance level for checking nonlinear and questionable results through retesting was undesirable (< 3 sigma). This indicates that our laboratory's performance was lower than studies conducted in Guangdong, China [42], Mysore, India [44] and Turkey [3] where performance levels were deemed desirable. The low performance of our laboratory may be due to a lack of verification process and insufficient awareness of the importance of critical value rechecking.

The results released without verification showed a low sigma value (0.7). Unverified results may be released as a result of such situations, which might be caused by workflow pressure, a lack of automated verification methods, or insufficient manpower. Patient safety and post‐analytical quality are jeopardized by incomplete result verification. Strict verification procedures should be followed before results are released, especially for critical and abnormal values, to avoid recurrence. To guarantee that all out‐of‐range findings are reviewed, laboratories should use automated verification systems with integrated delta checks and flagging features. Staff members should also receive training on the value of documenting and validating results. By putting these steps into practice, the overall post‐analytical quality performance will be improved and the reported results will be more reliable.

This study found that the sigma metric values for most analytes were satisfactory, with 8 analytes (ALP L2, uric acid, creatinine L2, HDL L2, total protein L1, total bilirubin L2, LDL, and creatinine kinase) exhibiting particularly strong performance (greater than 6 sigma). This aligns with previous research conducted in Addis Ababa, Ethiopia for (total bilirubin L2 and HDL L2) [48], China for (alkaline phosphatase L2, creatinine L2, HDL L2, LDL L2, total bilirubin L2 and uric acid) [49] and India for (ALP L2 and HDL L2) [50]. However, this finding was higher than studies conducted in Dessie, Ethiopia for (ALP L2, uric acid, creatinine L2, HDL L2, total protein L1, total bilirubin L2, and low density lipoprotein) [11], Addis Ababa, Ethiopia for (ALP L2 and total protein L1) [48], Ghana for (ALP L2, creatinine L2, HDL L2 and total protein L1) [51], Sudan for (creatinine L2) [52] and China for (total protein L1 and LDL L1) [49]. This discrepancy could be attributed to several factors, including variations in the analytical platform (Dimension EXL 200 Integrated Chemistry System was used in this study), quality of reagents and QC materials, study period, maintenance practices of the analyzers, and the competency of laboratory personnel.

Fifteen analytes (ALT, AST, ALT L1, creatinine L1, glucose L1, total cholesterol, triglyceride, HDL level 1, LDL, total protein L2, albumin, direct bilirubin L2, sodium L2, potassium L2 and chloride) demonstrated moderate performance, with sigma values ranging between 3 and 6. This result was consistent with studies conducted in Dessie, Ethiopia for (ALP L1) [11], Addis Ababa, Ethiopia for (total cholesterol, total protein L2, albumin, direct bilirubin L2, sodium L2, potassium L2 and chloride) [48], Sudan for (glucose L1) [52], Turkey for (creatinine L1, glucose L1, total cholesterol, triglyceride and albumin) [53], India for (glucose L1, total cholesterol, AST L2 and ALT L2) [50] and China for (glucose L1, total protein level 2, LDL L1 and chloride L1) [49]. However, this finding was higher than studies conducted in Dessie, Ethiopia for (ALT, AST, creatinine L1, glucose L1, total cholesterol, triglyceride, HDL L 1, LDL, total protein L 2 and albumin) [11], Addis Ababa, Ethiopia for (creatinine L1) [48], Ghana for (ALP L1, creatinine L1, high‐density lipoprotein L1, total protein L2, triglyceride, AST, sodium L2, potassium L2 and chloride) [51], Sudan for (creatinine L1and total cholesterol) [52], India for (ALT L1, direct bilirubin L2, AST L2 and creatinine L1) [50] and China for (sodium L2, potassium L2 and chloride) [49]. This reason may be due to differences in analytical techniques, internal quality control materials, proficiency testing bodies and manufacturers, the performance of the analyzer, the quality of reagents and QC materials, the study period, maintenance practices, and the competency of laboratory personnel.

On the other side, the 6 analytes (urea, glucose L2, total bilirubin level 1, direct bilirubin L1, sodium L1 and potassium L1) were found to be less than 3 sigma values. These findings were consistent with the studies performed on Dessie, Ethiopia for (urea, glucose L2, and total bilirubin L1) [11], Addis Ababa, Ethiopia for (urea and sodium L1) [48], Sudan for (urea) [52], Ghana for (urea and total bilirubin L 1) [51] and India for (urea and direct bilirubin L1) [50]. However, this finding is lower than the studies done in Addis Ababa, Ethiopia for (glucose L2, total bilirubin level 1, direct bilirubin L1, and potassium L1) [48], Sudan (glucose) [52], Turkey for (urea and total bilirubin L1) [53], India (glucose) [50], and China for (urea L1, glucose L2, total bilirubin level 1, direct bilirubin L1, sodium L1, and potassium L1) [49]. This discrepancy could be attributed to factors such as differences in analytical techniques, traceability of calibrators, instrumentation, quality control materials, pre‐analytical and analytical conditions, internal quality control materials, storage practices for reagents and QC materials, study period, maintenance practices, and competency of laboratory personnel.

This study revealed that the poor performance of certain analytes, as indicated by their low sigma values, was primarily driven by imprecision in the quality control (QC) process. However, this finding can have been influenced by a number of underlying causes. First, for enzyme‐based tests like urea and glucose, which are susceptible to reagent stability and storage conditions, shifts in the analytical mean may result from reagent lot‐to‐lot fluctuation and inadequate calibration frequency. Second, poor electrolyte assay performance (e.g., sodium and potassium) may be explained by electrode drift or poor instrument maintenance. Third, random error may have been exacerbated by operator‐related factors, such as disparities in experience, poor adherence to SOPs, and variations in pipetting technique. Lastly, assay precision may be further impacted by environmental factors including temperature and humidity variations in the analytical region. Several corrective and preventive measures are suggested in order to overcome these problems. Increased QC frequency and rigorous QC monitoring using multi‐rule Westgard procedures are necessary for low‐sigma analytes in order to identify analytical shifts early. Every time there are noticeable changes in QC means, reagent management should involve recalibration and verification of newly purchased reagent lots. Particularly for ion‐selective electrodes, regular equipment maintenance and calibration verification documentation will minimize measurement drift. To reduce operator‐dependent variability, there should also be focused staff training on reagent handling, equipment maintenance, and QC interpretation. Sustained performance improvement can also be supported by ongoing Levey‐Jennings chart monitoring and involvement in external quality assessment (EQA) programs. By putting these corrective actions into practice, it should be possible to improve the overall reliability of the testing procedure at Dessie Comprehensive Specialized Hospital and move underperforming assays into the desirable performance range (≥ 4σ). This finding aligns with previous research conducted in Dessie [11] and Addis Ababa, Ethiopia [48].

## 5 Strengths and Limitations of the Study

The strength of this study was that it assessed the TTP performance of the routine clinical chemistry parameters, electrolyte and hormonal tests using a relatively comprehensive checklist. Besides, the study included sigma metrics as the performance assessment tool for clinical chemistry laboratories in TTP and also assessed the performance of automated clinical chemistry machines. The limitation of this study was that hemolysis measurements were made by visual observation which may lead to interpersonal bias. Moreover, the study was carried out over a relatively short duration. Furthermore, laboratory samples that were collected outside of normal working hours were not included in the study.

## 6 Conclusion and Recommendation

### 6.1 Conclusion

This study concluded that a high frequency of clinical chemistry laboratory errors occurred. The majority of the errors were detected during the pre‐analytical phase, followed by the post‐analytical phase. Out of the pre‐analytical errors, incompleteness of the request forms and unsuitable samples constituted the majority. The sigma metric value of most analytes had marginal to world‐class performance. The clinical chemistry laboratory had poor overall sigma metric values (< 3 sigma). The laboratory had no guarantee on the quality of services provided.

### 6.2 Recommendations

We recommend that researchers do further studies with extended study periods involving many laboratory and health professionals. Besides, our recommendations for the Dessie Comprehensive Specialized Hospital are to provide training on laboratory quality service for laboratory professionals and non‐laboratory phlebotomists. In addition, we suggest that the hospital take immediate corrective action in TTP, prioritizing the pre‐analytical phase and considering sigma metric evaluation as one quality monitoring and improvement tool. Moreover, we recommend that professionals strictly follow SOP in all phases of the testing process. In general, the laboratory is better off implementing periodic and inclusive internal and external audits to take evidence‐based corrective measures.

## Author Contributions

Z.M. conceptualized and wrote the manuscript. T.G., M.A., A.G., E.E., B.E., and M.T. contributed to the design and implementation of the study, collected the data, performed the statistical analysis, performed the data interpretation, and drafted the manuscript; additionally, H.D., Y.K., and E.A. contributed to the data interpretation and drafting. All the authors reviewed the main manuscript.

## Ethics Statement

All the procedures were performed in accordance with the relevant guidelines and regulations. Ethical approval was obtained from the Ethical Review Committee of the College of Medicine and Health Science, Wollo University (reference number of: CMHS/138/24). The objective and purpose of the study were explained to the medical director, and a permission letter was obtained to collect the data. After providing an explanation of the possible benefits and risks written informed consent was obtained from the parents or legal guardians and staff. To ensure confidentiality of data, study subjects were identified using codes, and only authorized people had access to the collected data. Identified errors were linked to responsible people for better patient management and quality improvement.

## Consent

The authors have nothing to report.

## Conflicts of Interest

The authors declare no conflicts of interest.

## Data Availability

Data is provided within the manuscript. If additional data require, you will request the corresponding author.
